# Community ecology in 3D: Tensor decomposition reveals spatio-temporal dynamics of large ecological communities

**DOI:** 10.1371/journal.pone.0188205

**Published:** 2017-11-14

**Authors:** Romain Frelat, Martin Lindegren, Tim Spaanheden Denker, Jens Floeter, Heino O. Fock, Camilla Sguotti, Moritz Stäbler, Saskia A. Otto, Christian Möllmann

**Affiliations:** 1 University of Hamburg, Institute for Hydrobiology and Fisheries Science, Center for Earth System Research and Sustainability (CEN), KlimaCampus Hamburg, Große Elbstraße 133, Hamburg, Germany; 2 Centre for Ocean Life, National Institute of Aquatic Resources, Technical University of Denmark, Kemitorvet, Bygning 202, Kgs. Lyngby, Denmark; 3 Thünen-Institute of Sea Fisheries, Palmaille 9, Hamburg, Germany; 4 Leibniz-Centre for Tropical Marine Ecology, Fahrenheitstraße 6, Bremen, Germany; Department of Agriculture and Water Resources, AUSTRALIA

## Abstract

Understanding spatio-temporal dynamics of biotic communities containing large numbers of species is crucial to guide ecosystem management and conservation efforts. However, traditional approaches usually focus on studying community dynamics either in space or in time, often failing to fully account for interlinked spatio-temporal changes. In this study, we demonstrate and promote the use of tensor decomposition for disentangling spatio-temporal community dynamics in long-term monitoring data. Tensor decomposition builds on traditional multivariate statistics (e.g. Principal Component Analysis) but extends it to multiple dimensions. This extension allows for the synchronized study of multiple ecological variables measured repeatedly in time and space. We applied this comprehensive approach to explore the spatio-temporal dynamics of 65 demersal fish species in the North Sea, a marine ecosystem strongly altered by human activities and climate change. Our case study demonstrates how tensor decomposition can successfully (i) characterize the main spatio-temporal patterns and trends in species abundances, (ii) identify sub-communities of species that share similar spatial distribution and temporal dynamics, and (iii) reveal external drivers of change. Our results revealed a strong spatial structure in fish assemblages persistent over time and linked to differences in depth, primary production and seasonality. Furthermore, we simultaneously characterized important temporal distribution changes related to the low frequency temperature variability inherent in the Atlantic Multidecadal Oscillation. Finally, we identified six major sub-communities composed of species sharing similar spatial distribution patterns and temporal dynamics. Our case study demonstrates the application and benefits of using tensor decomposition for studying complex community data sets usually derived from large-scale monitoring programs.

## Introduction

Understanding the spatial and temporal dynamics of biotic communities containing large numbers of species represents a key challenge in ecology and is crucial to guide ecosystem management and conservation efforts. However, the interaction between the spatial distribution and the temporal dynamics of species assemblages is difficult to grasp and requires specific methods that account for the multidimensional nature of community data. In fact, community data are intrinsically multidimensional, because each sample taken in a given location at a given time can be described by the abundances of multiple species. Hence, data sets from monitoring programs with repeated sampling at multiple locations can be organized as a 3-dimensional array (i.e., 3rd-order tensor) with species, space and time being its three dimensions.

Common approaches in community ecology use “two dimensional” multivariate analysis methods such as Principal Component Analysis (PCA) or Correspondence Analysis to analyse community data [1,2]. Because most of the statistical methods are developed to analyse matrices, one of the three dimensions of community data is often sacrificed to reduce the 3D array into a 2D matrix. Depending on the aim of the study, scientists have been simplifying either the species assemblages into diversity indicators [3], the spatial distribution into barycentre coordinates [4], or the temporal dynamics by averaging over stable periods [5]. One way to keep the full information in 3D data sets is the extension of multivariate analysis to k-tables (such as STATIS [6]) and the simultaneous analysis of a sequence of paired ecological tables [7–9]. While the extension to k-tables is a clear improvement, which has found numerous applications among ecologists to study spatio-temporal patterns [10,11], the k-table approach considers one of the dimensions (often time or space) only as a repetition, restricting the results by the a-priori choice of the repetitive dimension and impeding the study of the interaction between time and space. Recently, other approaches have been developed to extend species distribution models to full communities, like the joint dynamic species distribution model [12,13] and the hierarchical modelling of species communities [14]. Multispecies distribution models are promising approaches, but strongly limited in size by the rapidly increasing number of parameters to be estimated. In contrast, multivariate approaches are free from parameters and can analyse data set with a large number of species in a high number of defined areas, for long-term time series. However, none of these multivariate methods can simultaneously study spatial and temporal dynamics, including the interaction between time and space across species assemblages which is needed for a comprehensive understanding of spatio-temporal changes of entire ecological communities [15].

Statistical tools able to investigate such multidimensional data sets were developed in the late 1960s within the fields of psychometrics [16]. Tensor decomposition (TD) methods (also called multiway multivariate analysis, tensor factorization, or high order principal component analysis) are becoming an essential tool for data mining and have been successfully applied within chemistry [17], neuroscience [18], bioinformatics [19], geophysics [20] and geospatial science [21]. The recent enthusiasm for TD fuelled by growing computing power and the emergence of big data [22], was followed by the development of new software [23,24]. Currently, multiple introductions and tutorials are available (e.g. [15,22,25]) and provide the basis for new applications using TD. An increased adoption of TD methods among ecologists could be beneficial because community data collected from large-scale ecological monitoring programs are inherently multidimensional (i.e. have more than 2 dimensions).

Here we demonstrate and promote the use of TD for disentangling spatio-temporal ecological dynamics using the North Sea demersal fish community as an informative example. The North Sea marine ecosystem has suffered from strong anthropogenic pressures [26], such as fisheries exploitation [27], and is already markedly impacted by climate change [28]. The need to manage the many commercially important fish populations providing highly valued ecosystem services [29] has resulted in a rigorous and internationally coordinated monitoring scheme in the North Sea [30]. The *North Sea International Bottom Trawl Survey* created a unique long-term (>30 years) data set covering multiple fish species abundance in time and space, which is openly available and provides an ideal basis for multiway analysis. Our study shows how TD can help (i) characterize the main spatio-temporal patterns of species assemblages, (ii) identify sub-communities that share similar spatial distribution and temporal dynamics, and (iii) reveal external drivers of change by applying additional correlation analyses and Monte-Carlo permutation tests.

## Materials and methods

### Tensor decomposition

**Fig 1:**
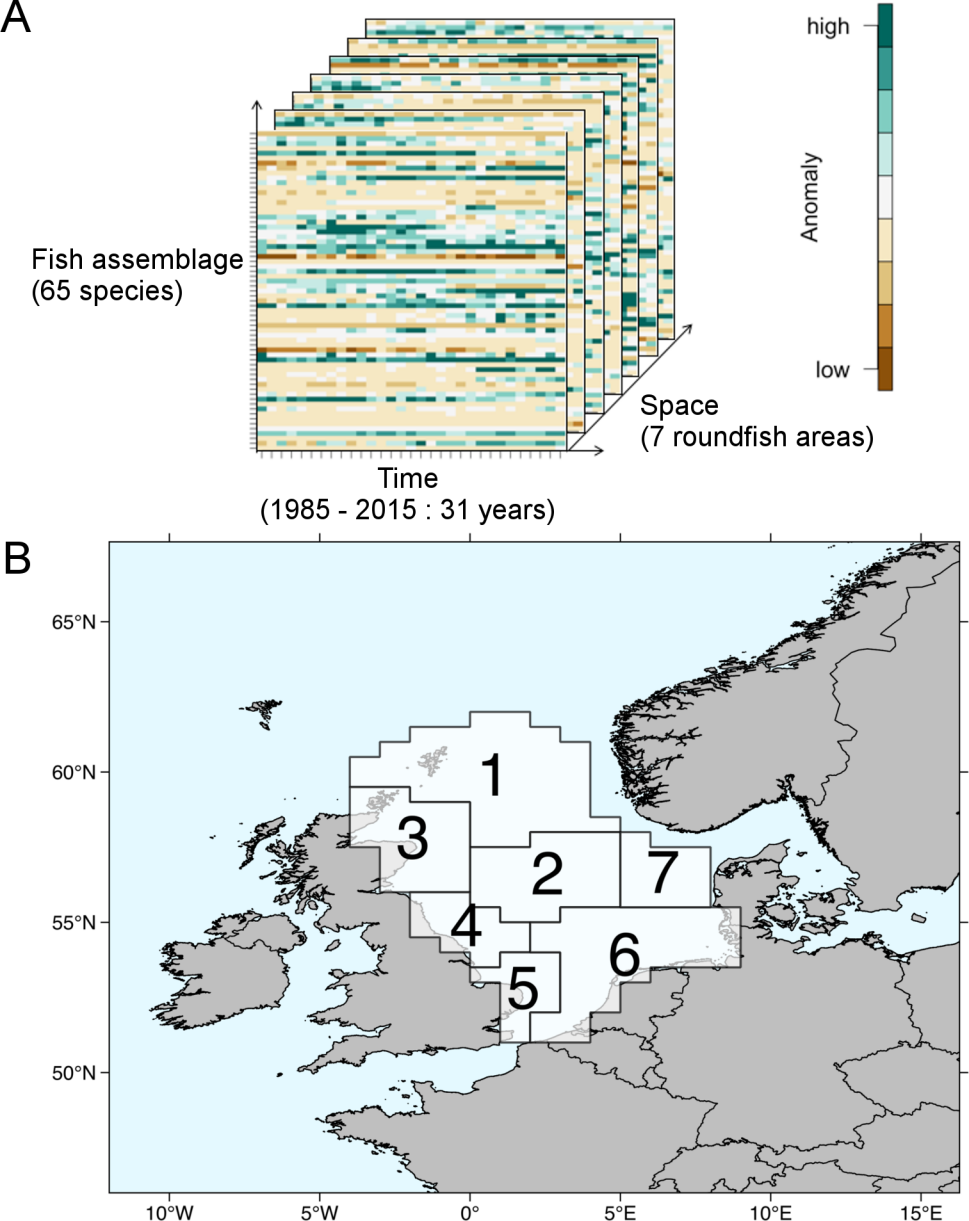
Presentation of the data set and its multidimensional nature. (A) Community data represented as a three-dimensional tensor. Each pixel represents the abundance level (relative to the average abundance of each species in the survey) of the fish species in the North Sea in its 3 dimensions: species, time and space. (B) Map of the study area showing the 7 predefined areas (called roundfish areas) which serve as the spatial scale of our study.

A tensor is a multidimensional array; a generalization of a matrix (two-dimensional table) in more than 2 dimensions. For example, the observed abundance of a species is associated with a given location and a given time. Community data are made of observations of abundances of multiple species (also referred as species assemblages), repeated in multiple areas and at different times. The data set can be seen as a three-dimensional (or third order) tensor with one dimension being the species taxa, a second dimension being the areas, and the third dimension being time (Fig 1A). To get reliable and complete time series, stations (locations of individual haul) are often aggregated to areas sharing similar features.

Tensor decomposition (TD) shares the same objectives of multivariate analysis, simplifying the original data set (here formatted as a tensor) and explaining the maximum proportion of the variance in the data set with a minimum number of components of lower dimensions. With this trade-off, TD reveals the main pattern (or information) within the data set and separates it from noise. Different methods of TD have been developed since the 1960s along with the development of multilinear algebra. Three methods are among the most popular: Tucker decomposition [16], canonical polyadic decomposition (also known by the acronyms CANDECOMP or PARAFAC, [31,32]) and Principal Tensor Analysis over k-modes (PTA) based on high order singular value decomposition [33]. Recent extensive reviews with detailed mathematical definitions and differences between these methods are available [15,25]. From a practical point of view, PTA offers an easy-to-interpret and robust method to decompose a tensor. Compared to the Tucker decomposition, PTA has the advantage of being independent of the dimensions of the desired solution (or core tensor). Compared to canonical polyadic decomposition, PTA has better explanatory power (due to its flexibility of having non-diagonal core tensor). The definition and algebra of PTA can be found in Leibovici and Sabatier [33]. We believe that its high similarity with the properties of the well-known PCA may facilitate its adoption by ecologists.

A PTA is completed following the same three steps as a PCA: (1) scaling, (2) selecting relevant components and (3) visual interpretation of the components with a biplot. First, the scaling and transformation of the original data is an essential preliminary step for the analysis of community data [34]. In the multidimensional context, the question is how to compute the distance between individuals and on which dimension(s) to center and/or scale the abundance values. The answer depends on the data set and the focus of the study. In our case, the abundance values were highly right skewed so we log(x+1)-transformed the abundance values and choose to use Euclidean distances as a basis for the PTA (similar to a PCA). Then, we centred (mean of 0) and scaled (standard deviation of 1) the abundance of each species to consider the rare and abundant species equally. Second, the dominant modes of variability, summarized in principal tensors (PTs), are selected with a scree plot, showing the percentage of the variance explained by each successive PT (S1 Fig). Similar to successive eigenvalues from PCA, a visual inspection of the scree plot indicates the number of significant PTs, i.e. the best trade-off between minimum number of PTs and the maximum percentage of variance explained [35]. Third, interpretation of PTs is made based on the projection of the dimensions on the selected PT (similar to a biplot in a PCA). PTA results in the simultaneous projection of the three dimensions (species, time and space) on simpler axis (PTs). The projection of time and space on the PT was plotted in a heatmap to represent the dominant spatio-temporal dynamics inherent in the data set.

However, the large number of species analysed in parallel renders the interpretation of the species projection derived by PTA difficult. Hence, we augmented the analysis by computing Euclidean distances between fish species from the projection of species scores on the PTs and subsequently conducted a Hierarchical Cluster Analysis (HCA) based on Ward’s criterion. We selected the significant number of groups from the HCA based on a graphical interpretation of the dendrogram. The robustness of the selected number of clusters was tested by comparison with the alternative K-Means Cluster Analysis (S3 Fig). Using cluster analysis, we derived a simplification of the dynamics of the multitude of individual species into fewer sub-communities sharing similar spatio-temporal patterns. We also used information about species’ traits, in terms of behaviour and life history, to characterize these sub-communities (data from [36]; S1 Table). We tested if sub-communities had significant differences in the distribution of traits with a Kruskal-Wallis test (for continuous traits) and Chi-square test (for qualitative traits).

### Example data set–the demersal fish assemblage of the North Sea

Abundance data of the North Sea demersal fish assemblage were compiled by the ICES (International Council for the Exploration of the Sea) Database for Trawl Surveys (DATRAS; http://datras.ices.dk/; data downloaded on the 16^th^ of February 2016). Data were collected by the *North Sea International Bottom Trawl Survey* [30], an international effort to monitor fish populations and communities. Each contributing research vessel applies a standard otter trawl as sampling gear. Individual hauls are standardized to catch per unit effort, which represents the average catch per unit of time of trawling. To assure representative sampling of the fish community, at least two hauls are regularly conducted in pre-specified spatial rectangles (ICES statistical rectangles) of one degree longitude and 0.5 degree latitude each [30].

For our analysis, we only used data collected during the first quarter (January-March) of the year, in order to avoid seasonal bias and benefit from the longest time series available (31 year continuous period from 1985 to 2015). We investigated spatio-temporal changes of the fish community on the scale of the seven predefined areas (called roundfish areas by ICES, RAs; Fig 1B) which sub-divide the North Sea based on ecological aspects of the fish fauna, including spawning, feeding and species composition [3,37]. Hence, our data set represents reliable relative annual abundance estimates per RA based on the aggregation of at least 16 hauls. Our approach sacrifices spatial information collected on the smaller rectangle basis (ICES statistical rectangles) for a better estimation of the abundance on each spatial unit. Nevertheless, conducting the analysis on a higher spatial resolution resulted in similar outcomes (S2 Fig).

We conducted pre-processing checks for misnaming or misidentification of species, removal of non-fish organisms and pelagic fish species that are not representatively sampled by the gear [38]. Furthermore, we excluded sporadic species that occurred less than once every year in at least one area. By this procedure, we removed 50% of the total number of species recorded. However, these species amounted to less than 1% of the total abundance. Our final data set contained annual abundance expressed in catch per unit effort of 65 individual species for the period 1985 to 2015 averaged over the seven RAs. We log(x+1)-transformed and then standardized the data (zero mean and sd of 1) to reduce the skewness and to scale rare and abundant species equally. Finally, we organized the data for the statistical analysis in an array of three dimensions, i.e. species, space and time, which we refer to as a tensor in the following text (Fig 1A).

Information about the biological characteristics of the species (maximum length, trophic level and biogeography) were extracted from Engelhard et al. [36]. Boreal fishes are species that extend north to the Norwegian Sea and Icelandic waters. Lusitanian fishes tend to be abundant from the Iberian Peninsula to as far north as the British Isles and the central North Sea. Atlantic species are species widespread in the North Atlantic.

All data analyses were performed with the statistical software environment R [39]. The PTA method is implemented in the R package PTA-k [23] and a tutorial (containing script and data) explaining TD on fish assemblages is openly available on GitHub (https://github.com/rfrelat/Multivariate2D3D, 10.5281/zenodo.831739).

### Environmental conditions and fishing pressure influences on spatio-temporal community patterns

We explored the effects of natural and anthropogenic drivers known to affect fish distribution patterns in the North Sea [29], specifically depth, local hydrographic conditions, primary productivity, large-scale climate indices and fishing pressure. Depth was retrieved from the General Bathymetric Chart of the Oceans, (GEBCO 2014 grid, www.gebco.net) and averaged per subdivision. Local hydrographic conditions were represented by bottom and surface temperatures and salinities derived from optimally interpolated observations of the North Sea [40]. Chlorophyll *a* (Chl) concentration (as proxy for primary production) was estimated from GlobColour (http://globcolour.info), a product developed, validated, and distributed by ACRI-ST, France [41]. The oceanographic data set and GlobColour are provided with a monthly time step and at high spatial resolution (respectively 0.2° and 1km). We spatially averaged these values over the RAs and derived three temporal indices from the 12 monthly values: an annual index (averages over 12 months, labelled with the subscript _AN_), a first quarter index (average values over January-February-March, labelled with the subscript _Q1_) and a seasonality index (difference between the maximum and minimum monthly value, labelled with the subscript _var_). The annual and the seasonality indices were compared to the fish abundance estimated in the first quarter of the following year. We restricted the number of temporal indices by considering a maximum lag of 1 year between the possible drivers and responses in fish abundance, which represents recruitment success of most species in the North Sea.

Large-scale climate conditions were represented by the Atlantic Multidecadal Oscillation (AMO, [42]), as well as the North Atlantic Oscillation (NAO, [43]) index. The two indices are known to affect the ecosystems of the North Atlantic and adjacent seas [44,45]. The NAO indicates high frequency (7–25 years) atmospheric variation, whereas AMO is a low frequency multidecadal (60 years) variation of the sea surface temperature. Time-series on both indices were derived from the climate indices platform of the Earth System Research Laboratory: http://www.esrl.noaa.gov/psd/data/climateindices/list/. Fishing effort as an index of exploitation pressure was estimated from a data set provided by the Scientific, Technical and Economic Committee for Fisheries of the European Commission (https://datacollection.jrc.ec.europa.eu/data-dissemination). Annual fishing efforts (in hours per ICES rectangle per year and per gear type) are available from 2003 onwards. We followed the recommendation by Engelhard et al. [27] to consider beam and otter trawl effort separately.

Potential external drivers were identified through correlation analysis with the derived PTs, as well as with the spatio-temporal dynamics of the sub-communities (represented by the spatio-temporal distribution of species aggregated by cluster). For drivers that can be defined in time and space (e.g. hydrography and fishing effort), the relationships between the spatio-temporal matrices were tested using the RV coefficient, a generalization of the Pearson correlation coefficient for matrices, and applying a Monte-Carlo permutation test with 5000 permutations [46]. For 1-dimensional drivers defined only in time (e.g. climatic indices) or space (i.e. depth), Pearson correlation coefficients were computed. To account for the autocorrelation inherent in the time-series affecting significance levels, p-values were calculated from 5000 random time series with similar first order autocorrelation (AR1). Eventually, the p-values were adjusted for multiple testing to correct false discovery rates following a method suggested by Benjamini and Yekutieli [47].

## Results

### Spatio-temporal dynamics of fish assemblages in the North Sea

PTA decomposed the initial tensor, i.e. the array of fish abundance in the three dimensions–species, space and time—into the dominant modes of variability, summarized in PTs. Based on a visual interpretation of the scree plot, we identified four significant PTs, which in total explained 43.2% of the variability in the North Sea demersal fish data set (S1 Fig). We evaluated the significance of our results by performing a PTA on the same tensor, but with its values randomly shuffled. This analysis explained only 6% of the total variability, suggesting our decomposition to reliably capture the main patterns in our data set. For simplicity and analogy with the more common PCA, we renamed the significant PTs with a number according to the decreasing order of variability explained, which differs from the labelling provided by the software used. The full results of the PTA (i.e., the output of the PTAk package) are provided in S1 Fig.

**Fig 2:**
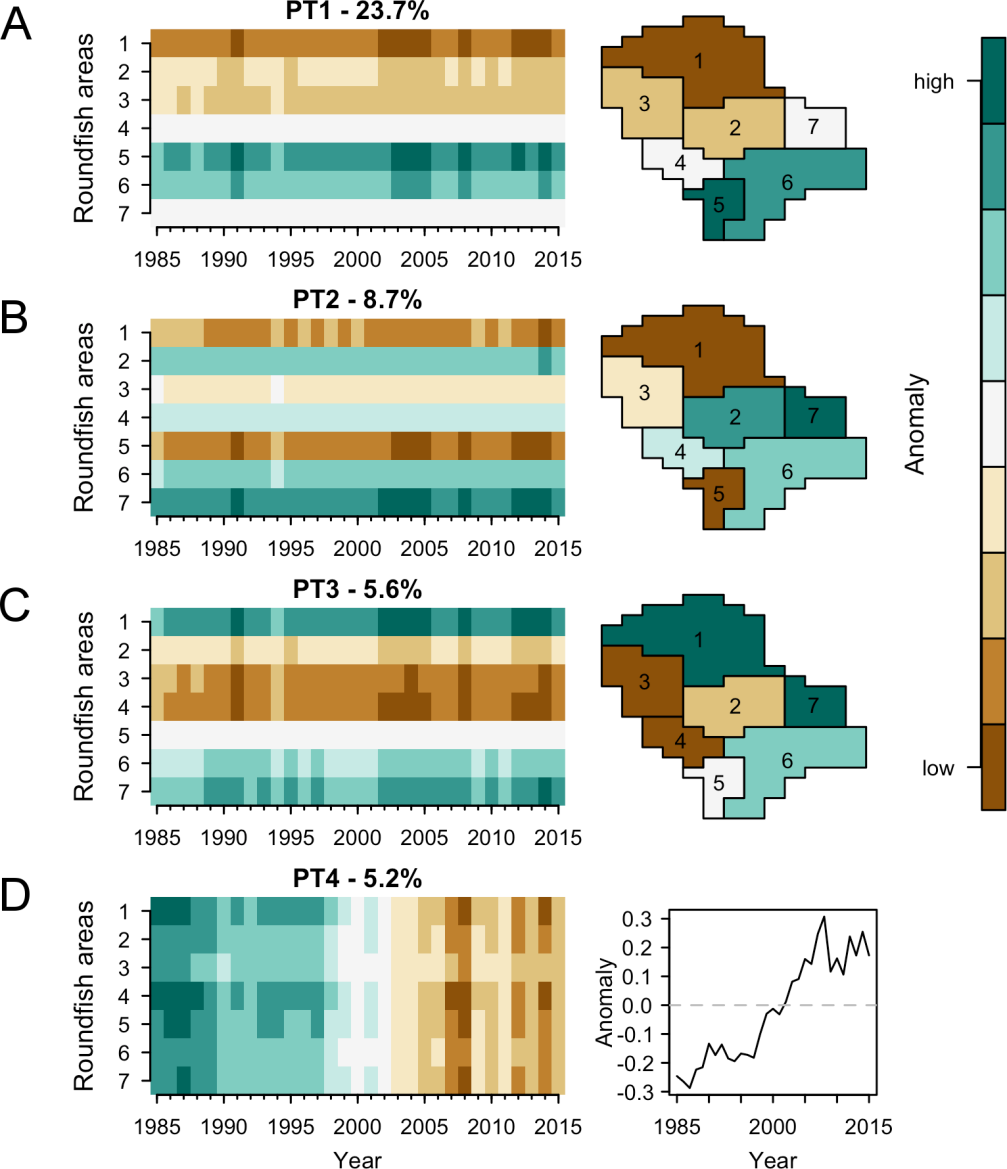
Results of the principal tensor analysis with 4 principal tensors (PT) explaining together 43% of the total variability in the North Sea fish assemblage. (A) PT1 showed a spatial gradient in species abundance from North to South. (B) PT2 showed the difference in abundance between strongly localized species (either in the North or the South) and species living in the central part of the North Sea. (C) PT3 showed the abundance difference between species in the West and East of the North Sea. (D) PT4 showed a temporal trend in species abundance.

Our results showed that the spatial structure of the fish assemblage explained a larger proportion of the variability in the data set compared to temporal variability. Spatial structure is represented by three PTs that together explained 38% of the total variability. The temporal pattern on the other hand is represented by only one PT and explained 5.2% of the total variability. We used heatmaps with time and space on the x- and y-axes respectively, to visualize the four main patterns found (Fig 2). PT 1–3 (Fig 2A–2C) revealed strong differences between RAs displayed by homogeneity in the abundance levels over time (i.e. homogeneity in row colours). PT4 (Fig 2D) represented a component of temporal variation that is homogeneous in space (i.e. homogeneity in column colours).

PT1 explained 23.7% of the variability in the data set and discriminated the northern (RA 1, 2 and 3) and southern parts (RA 5 and 6) of the North Sea (Fig 2A). Correlation analysis revealed significant relationships of PT1 with Chl (Chl_Q1_, p-value = 0.03), the seasonality of sea bottom temperature (SBT_var_, p-value = 0.03) and of sea bottom salinity (SBS_var,_, p-value = 0.03) (Table 1 and S2 Table). Moreover, PT1 was correlated with sea bottom salinity (SBS_Q1_, p-value = 0.07) and depth (p-value = 0.07). Therefore, PT1 highlighted the differences of fish species living in the southern part of the North Sea, a shallow area with high primary production and pronounced seasonality in bottom temperature and salinity, compared to fish species living in the northern part, a deeper area with lower primary production and lower seasonal variations in temperature and salinity. In other words, the analysis revealed a strong north-south gradient in the composition of the North Sea fish community corresponding to a strong gradient in geography, hydrography and biological productivity.

**Table 1:** Correlation analysis to identify environmental influences on spatio-temporal community patterns.

	Tensor Decomposition				Clusters					
	PT1	PT2	PT3	PT4	Southern	Northern	NW Inc	SE Inc	Increasing	Decreasing
SST_Q1_	0.47	0.24	0.4	0.03	0.25	0.21	0.73	0.93 *	0.44	0.07
SST_var_	0.51	0.26	0.28	0.02	0.24	0.27	0.65	0.96 *	0.49	0.05
SBT_Q1_	0.64	0.39	0.05	0.05	0.32	0.56	0.39	0.89 *	0.59	0.13
SBT_var_	0.94 *	0.01	0.03	0.03	0.81 °	0.45	0.61	0.67	0.06	0.46
SSS_Q1_	0.68	0.20	0.14	0.10	0.40	0.49	0.49	0.84 °	0.39	0.18
SSS_var_	0.08	0.41	0.31	0.18	0.09	0.07	0.20	0.39	0.48	0.27
SBS_Q1_	0.84 °	0.06	0.02	0.03	0.56	0.73 °	0.29	0.61	0.18	0.32
SBS_var_	0.90 *	0.13	0.05	0.04	0.67	0.55	0.54	0.79 °	0.25	0.36
Chl_Q1_	0.92 *	0.08	0.02	0.00	0.97 °	0.35	0.56	0.45	0.01	0.68
Chl_var_	0.83 °	0.15	0.04	0.02	0.97 *	0.26	0.55	0.34	0.04	0.74
Otter	0.52	0.31	0.12	0.23	0.19	0.88	0.05	0.46	0.47	0.05
Beam	0.64	0.01	0.06	0.05	0.52	0.28	0.52	0.55	0.08	0.20
AMO	0.40	-	-	0.74 °	0.70	-0.72 °	0.74 °	0.74 °	0.74 °	-0.74 °
NAO	0.17	-	-	-0.06	-0.01	0.03	-0.06	-0.06	-0.06	0.06
Depth	0.88 °	0.36	-0.27	0.30	0.66	-0.96 *	-0.38	0.75	-0.52	-0.5

Correlation coefficients between drivers (in rows) and spatio-temporal dynamics of the North Sea fish assemblages (in columns). Pearson correlation coefficients and RV coefficients were calculated for 1- dimensional (e.g. climatic indices, depth) and 2 dimensional (e.g. hydrography and fishing effort) drivers, respectively. The subscript ‘Q1’ represents the first quarter of the year, ‘var’ indicates the seasonality of the previous year, i.e. the difference between the minimum and the maximum monthly values. PTs are the principal tensors found by the Principal Tensor Analysis; the spatio-temporal dynamics of the clusters were represented by the projection of their barycentre on the PTs.

Adjusted p-values to correct for false discovery rates in multiple testing were computed and correlation significance is indicated by

‘°’ p<0.1.

‘*’p <0.05.

PT2 and PT3 explained 8.7 and 5.6% of the variability in the data set, respectively. PT2 showed the connectivity of fish communities to other seas and opposes the Atlantic entrance of the North Sea (toward the Norwegian Sea, RA 1, and the English Channel, RA 5) to the Baltic Sea entrance (RA 7) (Fig 2B). PT3 discriminated the western (RA 3 and 4) and eastern NS (RA 1 and 7) (Fig 2C). PTs 2 and 3 were tensors with a temporal mode associated to PT1, i.e. they shared the same temporal components. PTs 2 and 3 were uncorrelated with environmental conditions and fishing pressure (Table 1). PT4 displayed the main temporal trend in the fish community and discriminated parts of the community continuously decreasing in abundance compared to those continuously increasing over the last 30 years (Fig 2D). The trend shown by PT4 was correlated with the AMO (p-value = 0.07, Table 1).

### Characteristic sub-communities of North Sea fish species

**Fig 3:**
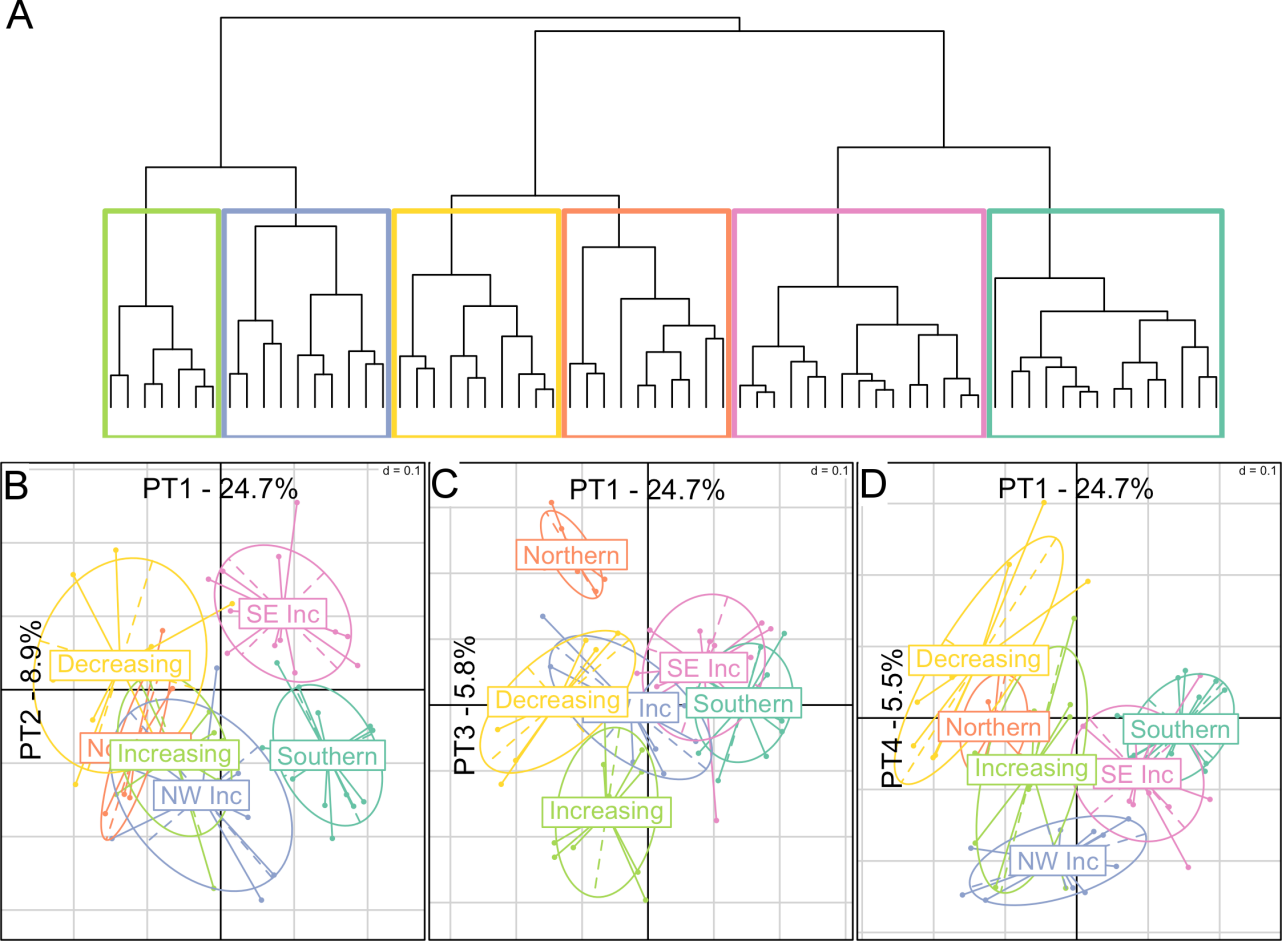
Classification of fish species based on their spatio-temporal dynamics. (A) Dendrogram of the Hierarchical Agglomerative Clustering and the separation of 6 clusters. (B-D) Clusters represented on the different principal tensors (PTs), with x-axis showing PT1 projections, and y-axis showing (B) PT2, (C) PT3 and (D) PT4 projections.

**Fig 4:**
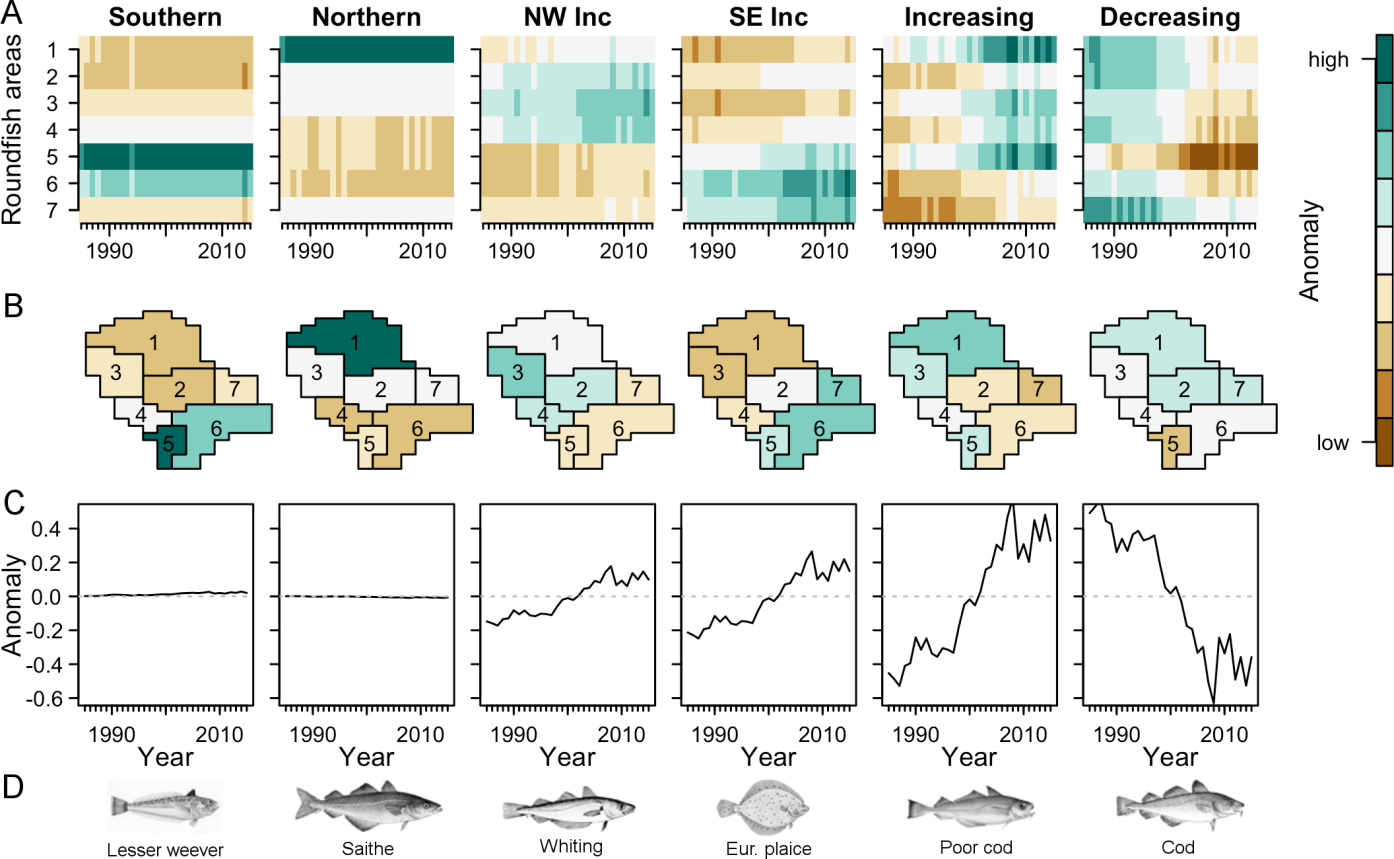
Main characterization of the sub-communities. (A) Spatio-temporal abundance, represented in a heatmap with time on the x-axis and space on the y-axis. (B) Spatially average abundance of the clusters in the roundfish areas. (C) Temporal average of the abundance per cluster. (D) Illustrations of fish species with the highest abundance in the respective cluster (images from FAO and Wikimedia).

The projection of the fish species on the four PTs was used to cluster species according to their spatio-temporal dynamics (Fig 3A). The dendogram indicated six clusters of species confirmed by the scree test (S3 Fig). The six clusters were projected separately on the four PTs (Fig 3B–3D). Two clusters (*Southern* and *Northern*) had strong spatial patterns and no temporal trend, while two clusters (*North-West Increasing* and *South-East Increasing*) had a strong spatial pattern and a weak temporal trend. The two remaining clusters (*Increasing* and *Decreasing*) were characterized by a temporal pattern (Fig 4). In the following, clusters of species are referred to as sub-communities and we labelled them according to their spatio-temporal characteristics and characterized them through key species (identified by having the highest average abundance and represented by drawings in Fig 4). The full species list and their assignments to the identified sub-communities is given in S1 Table.

The *Southern* sub-community consisted of 14 fish species, among them lesser weever (*Echiichthys vipera*) and sole (*Solea solea*) with a distribution concentrated in the southern NS (RAs 5 and 6) and very low abundance in the northern RAs 1 and 2 (Fig 4). The *Southern* community was positively and significantly correlated with the Chl concentration and its seasonality (Chl_Q1_ and Chl_var_; p-value = 0.07 and 0.03 respectively, Table 1) and mean annual sea bottom temperature of the previous year (SBT_an_; p-value = 0.04, S2 Table). The *Northern* community consisted of a cluster of 10 fish species, among them saithe (*Pollachius virens*), with high abundance in the North (RA 1) and very low occurrence in the southern RAs 4 and 6. The *Northern* community was negatively correlated with depth (p-value = 0.03) and had a weak positive relationship with sea bottom salinity (SBS_Q1_, p-value = 0.07).

The *North-West increasing* sub-community was a very heterogeneous cluster composed of 15 fish species, including whiting (*Merlangius merlangus*), haddock (*Melanogrammus aeglefinus*), dab (*Limanda limanda*) and norway pout (*Trisopterus esmarkii*). The high heterogeneity of the sub-community resulted in weak temporal and spatial pattern, which appeared to be uncorrelated with environmental conditions and fishing pressure (Table 1). A positive temporal trend was observed for the *South-East increasing* sub-community, which consisted of 12 fish species, among them plaice (*Pleuronectes platessa*), concentrated in RAs 6 and 7. The *South-East increasing* sub-community was significantly correlated with sea bottom temperature (SBT_Q1_, p-value = 0.03) and with sea surface temperature and its seasonality (SST_Q1_ and SST_var_, p-value = 0.04 and 0.03 respectively).

The *Increasing* sub-community was a cluster composed of 11 species, among them poor cod (*Trisopterus minutus*) and hake (*Merluccius merluccius*) with a positive temporal trend and a weak spatial preference (Fig 4) for the entrance of the Atlantic Ocean (RA 1 and 3) or the English Channel (RA 5). The *Decreasing* community was composed of a cluster of only 4 fish species, among them cod (*Gadus morhua*) and starry ray (*Amblyraja radiata*) characterized by a strong decreasing trend during the past 30 years. The two sub-communities *Increasing* and *Decreasing* were uncorrelated with environmental conditions and fishing pressure (Table 1).

**Fig 5:**
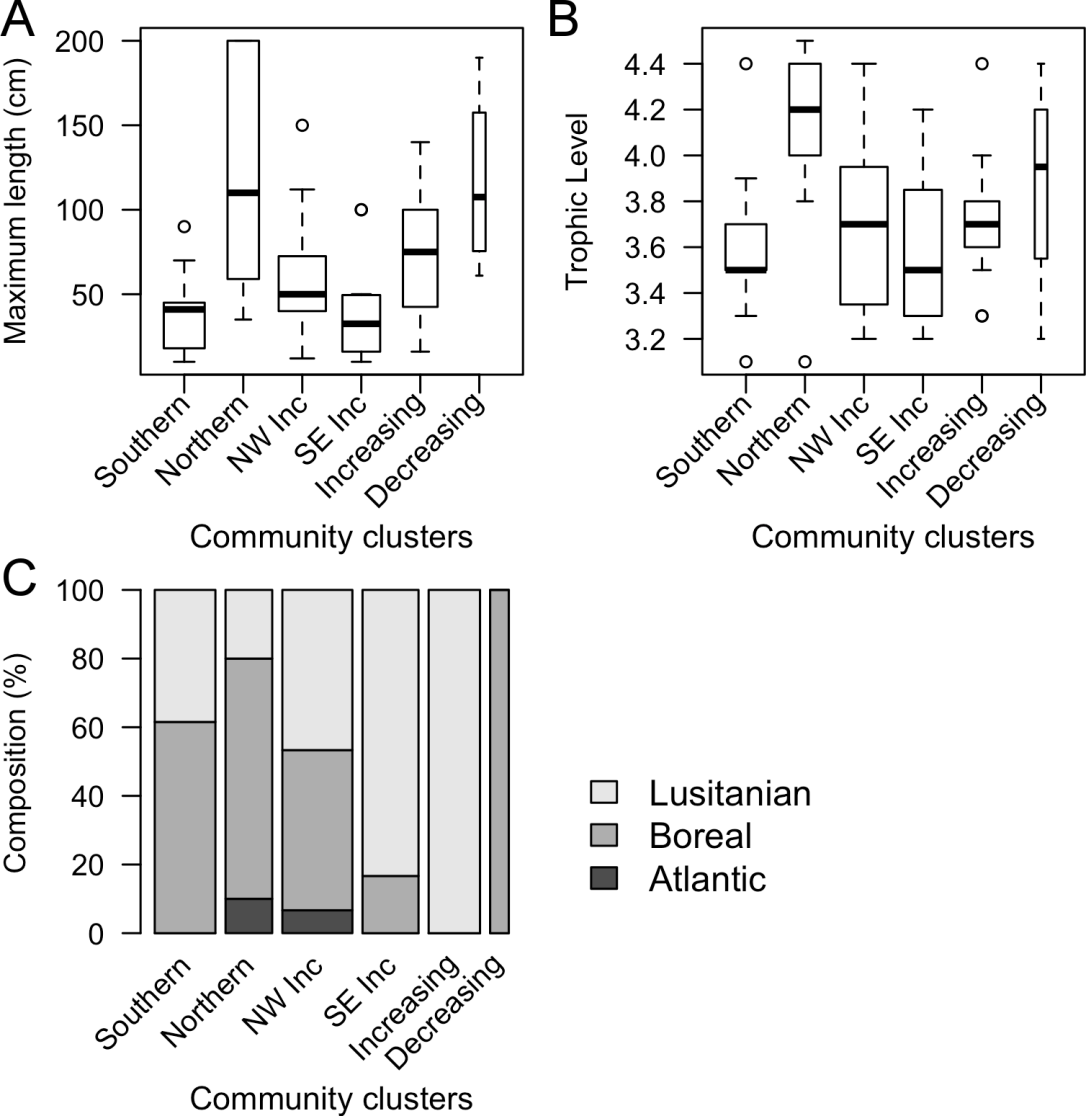
**Biological characteristics (A: maximum length, B: trophic level and C: biogeography) of the spatio-temporal clusters**. Widths of the boxplot (A-B) and the bars (C) are proportional to the number of species per cluster.

Finally, we investigated the biological characteristics of the six sub-communities described above by comparing the traits of species classified into each sub-community. The distribution of biological traits significantly reflected the north-south division of the fish sub-communities (Fig 5). Fish species were on average larger (significant difference, p-value = 0.001) in the *Northern* sub-community (median of 110 cm) and the *Decreasing* sub-community (107.5 cm), compared to fish in the *Southern* sub-community (41 cm) and *South-East increasing* sub-community (32.5 cm). The same separation was evident in the trophic level of the fish species (Fig 5B, p-value = 0.04). The *Northern* and the *Decreasing* sub-community had a higher trophic level (on average 4.1 and 3.9, respectively) while the *Southern* sub-community and *South-East increasing* sub-community displayed lower average trophic levels (3.6 and 3.6, respectively). Furthermore, biogeography was a good indicator of the main temporal trends in the North Sea fish community (Fig 5C, p-value = 0.002). The *Increasing* and *South-East increasing* sub-community were mainly composed of Lusitanian species, while the *Decreasing* cluster contained only boreal species. The latter division indicated the climatic influence on the temporal development of the North Sea fish community.

## Discussion

We demonstrated the use of TD, an integrative statistical analysis for studying multi-dimensional data sets, typically collected by large-scale ecological monitoring programs. Here we analysed a multi-decadal data set on the spatial distribution of 65 demersal North Sea fish species to better understand the spatial structure and recent temporal changes in the species assemblages. Our study shows that TD is able to identify strong and persistent spatial structure in the fish community while simultaneously identifying strong temporal changes in abundance.

The first main outcome of our study was the identification of a strong and stable spatial structure of the fish community into a *Northern* and a *Southern* sub-community. Correlation analysis explained this structure by differences in depth, primary production levels (represented by Chl concentrations), as well as the seasonality of temperature and salinity conditions. The demonstration of two very different sub-systems confirms earlier investigations in the area [3,48]. To a lesser degree, our analysis revealed a west to east gradient in community dynamics (PTs 2 and 3), which despite insignificant correlations with the explaining variables used, is likely related to the transition zones to the open Atlantic Ocean (PT2) and to the Baltic Sea (PT3). The spatial structuring revealed by our TD approach is robust to using biomass instead of abundance and especially to the spatial scale applied (S2 Fig). A higher resolution, i.e. on a statistical rectangle basis and therefore sacrificing sample sizes and adding noise in the estimated abundance, revealed the same spatial community structure as shown with the relatively coarse spatial scale of the RAs.

A second main outcome of our study was that despite the strong and predominant spatial structure our method was able to identify strong temporal changes in the fish community. Although explaining only a comparatively small fraction of the overall variability in the data set, this temporal trend indicates changes in community dynamics with a strong turning point around the late 1990s and the early 2000s. Correlation analysis indicated this change to be at least partly climate driven since it was correlated to the low frequency temperature variability of the AMO. The results confirm the importance of the recent positive anomaly phase of the AMO for ecosystem dynamics in the North-East Atlantic shown in earlier studies targeting single fish species or only small parts of the fish community alone [49,50], lower trophic level dynamics [51] and multi-trophic ecosystem changes [45,52]. Our correlation analysis showed no significant correlation with fishing effort, although there is undoubtedly a high impact of fishing on many commercially important species [27]. We attribute this result to the length of the time-series of fishing effort beginning only in 2003. However, if the low and non-significant correlations with fishing effort are a result of data shortage or are a result of the TD methodology remains to be seen in future studies.

We used hierarchical cluster analysis on the PT projection to identify sub-communities that group species sharing similar spatio-temporal dynamics. We verified the internal consistency of these six sub-communities by investigating the distribution of biological traits within the identified clusters. This approach revealed meaningful results, showing that the *Northern* sub-community is composed of mainly boreal species with larger sizes and higher trophic levels compared to the smaller Lusitanian species residing primarily in the southern North Sea. Interestingly, the homogeneity in biological traits that we found for the clusters based on spatio-temporal dynamics supports the theory that organisms sharing similar traits exhibit similar dynamics [5,36]. However, future analyses would benefit from using additional biological characteristics.

As with any statistical approach, the ability of the method applied here is limited by the quality and amount of data available. For example, the data set used covers the period 1985–2015, not including the stable period before the regime shift occurring in the North Sea during the late 1980s [53,54]. Including this period of change would likely increase the importance of the temporal component (represented by PT4) compared to the spatial components (PTs 1–3) by increasing the range of variability in species abundance fluctuations. Spatial limitations, however, mean that we cannot track fish species that move out of the study area. Moreover, limited sample sizes forced us to conduct the analysis on the scale of the seven RAs which may mask fine-scale spatial variability. However, as mentioned previously, we performed an additional analysis at the spatial scale of 168 statistical rectangles and found similar results (S2 Fig).

Overall, the results of our study have implications for the design of future modelling studies with respect to spatial structure and trophic group composition of fish assemblages, for example in food web models [55]. Similarly, our results can readily inform future ecosystem-based management approaches that are multi-species or community-based compared to the prevailing single-species approaches [56,57]. For example, PTA could be used to define species assemblages based on in-situ data or, with a finer spatial scale, to define areas for management based on ecology rather than current political boundaries. Furthermore, we argue and conclude that multiway statistical approaches accounting for multiple dimensions of community data are fruitful and ready for uptake in community ecology and macroecology. Ecosystems and the species they contain vary both in time and in space. Classical two-way analyses simplify this information, which is inherently three dimensional, and therefore cannot investigate the multiple interactions between these dimensions [15]. Methods such as the one applied here reveal these multidimensional patterns and provide a promising tool for knowledge discovery in large-scale data sets derived from modern ecological monitoring programs.

## Supporting information

S1 Table Biological characteristics of species, ordered by cluster. Information about the biogeography, the trophic level (TL), the maximum length (Lmax) are from Engelhard *et al*., 2011. Average Catch per Unit Effort (av CPUE) are calculated from the data itself.
(PDF)

S2 Table Full correlation coefficient table. Table of Pearson and RV correlation coefficient (c) with p-value (p) and adjusted p-value (ap).
(PDF)

S1 Fig Results of the Principal Tensor Analysis. Output of the *PTA-k* R-package (top) and selection of the four principal tensors (PTs) based on the scree-plot (bottom).
(PDF)

S2 Fig Robustness analysis to data transformation and spatial scale. TD computed with (A) the method presented in the manuscript (abundance expressed in number/hour, at the scale of roundfish areas), (B) abundance expressed in biomass (catch per unit effort, expressed in kg/hour) and (C) a finer spatial resolution, at the scale of ICES rectangle (and abundance in number/hour). The three decompositions are similar, with 4 significant PT. The three first PTs show strong spatial patterns, while the PT4 shows a trend in time series.
(PDF)

S3 Fig Clustering analysis of the fish species realised with K-means algorithm. (PDF)
