# Correction: Community ecology in 3D: Tensor decomposition reveals spatio-temporal dynamics of large ecological communities

**DOI:** 10.1371/journal.pone.0196353

**Published:** 2018-04-19

**Authors:** Romain Frelat, Martin Lindegren, Tim Spaanheden Dencker, Jens Floeter, Heino O. Fock, Camilla Sguotti, Moritz Stäbler, Saskia A. Otto, Christian Möllmann

The third author's name is spelled incorrectly. The correct name is: Tim Spaanheden Dencker. The correct citation is: Frelat R, Lindegren M, Dencker TS, Floeter J, Fock HO, Sguotti C, et al. (2017) Community ecology in 3D: Tensor decomposition reveals spatio-temporal dynamics of large ecological communities. PLoS ONE 12(11): e0188205. https://doi.org/10.1371/journal.pone.0188205
