# Supplementary material for: Community ecology in 3D: Tensor decomposition reveals spatio-temporal dynamics of large ecological communities
Source: PLoS One. 2017 Nov 14;12(11):e0188205. doi: 10.1371/journal.pone.0188205 (PMC5685633; doi:10.1371/journal.pone.0188205)
Supplement: S1 Table — Information about the biogeography, the trophic level (TL), the maximum length (Lmax) are from Engelhard et al., 2011. Average Catch per Unit Effort (av CPUE) are calculated from the data itself. (PDF) [file pone.0188205.s001.pdf]

S2 Table. Biological characteristics of species, ordered by cluster. Information about the biogeography, the trophic level (TL), the maximum length (Lmax) are from Engelhard *et al.*, 2011. Average Catch per Unit Effort (av CPUE) are calculated from the data itself.

	Species	Common name	Biogeography	TL	Lmax (cm)	av CPUE
Southern	*Agonus cataphractus*	Pogge (armed bullhead)	Boreal	3.4	21	5.0
	Ciliata mustela	Five-bearded rockling	Boreal	3.5	45	0.8
	Echiichthys vipera	Lesser weever fish	Lusitanian	4.4	15	15.2
	Enchelyopus cimbrius	Four-bearded rockling	Boreal	3.5	41	3.2
	Gaidropsarus vulgaris	Three-bearded rockling	Lusitanian	3.3	43	0.0
	Liparis liparis	Sea snail	Boreal	3.6	18	2.2
	Liparis montagui	Montagu's seasnail	Boreal	3.5	10	0.1
	Myoxocephalus scorpius	Bullrout (father lasher)	Boreal	3.9	60	2.9
	Pholis gunnellus	Butterfish (gunnel)	Boreal	3.5	25	1.0
	Raja clavata	Thornback ray	Lusitanian	3.8	90	2.5
	Solea solea	Sole	Lusitanian	3.1	70	2.9
	Taurulus bubalis	Sea scorpion	Boreal	3.6	18	0.7
	Trisopterus luscus	Bib	Lusitanian	3.7	45	3.8
Northern	*Brosme brosme*	Tusk	Boreal	4	100	0.0
	Glyptocephalus cynoglossus	Witch	Boreal	3.1	60	0.9
	Helicolenus dactylopterus	Bluemouth redfish	Atlantic	3.8	44	0.2
	Hippoglossus hippoglossus	Halibut	Boreal	4.5	200	0.0
	Lepidorhombus whiffiagonis	Megrim	Lusitanian	4.2	59	0.8
	Lophius piscatorius	Anglerfish	Lusitanian	4.4	200	0.6
	Molva molva	Common ling	Boreal	4.3	200	0.4
	Pollachius pollachius	Pollack	Boreal	4.2	130	0.3
	Pollachius virens	Saithe	Boreal	4.4	120	11.6
	Sebastes viviparus	Norway haddock	Boreal	4	35	0.2
NW Inc	*Callionymus lyra*	Common dragonet	Lusitanian	3.3	32	4.0
	Eutrigla gurnardus	Grey gurnard	Lusitanian	3.6	50	200.4
	Hippoglossoides platessoides	Long rough dab	Boreal	3.7	50	93.7
	Leucoraja naevus	Cuckoo ray	Lusitanian	3.9	70	1.0
	Limanda limanda	Dab	Boreal	3.3	40	619.4
	Melanogrammus aeglefinus	Haddock	Boreal	4.1	112	685.7
	Merlangius merlangus	Whiting	Lusitanian	4.4	70	1353.5
	Microstomus kitt	Lemon sole	Boreal	3.3	45	10.6
	Mullus surmuletus	Striped red mullet	Lusitanian	3.4	40	1.0
	Mustelus mustelus	Smoothhound	Lusitanian	3.8	150	0.1
	Myxine glutinosa	Hagfish	Atlantic	3.5	45	0.0
	Phrynorhombus norvegicus	Norwegian topknot	Boreal	4	12	0.3
	Scophthalmus rhombus	Brill	Lusitanian	3.8	75	0.1
	Squalus acanthias	Spurdog	Boreal	4.3	105	1.5
	Trisopterus esmarkii	Norway pout	Boreal	3.2	26	1828.5
SE Inc	*Arnoglossus laterna*	Scaldfish	Lusitanian	3.6	20	0.7
	Buglossidium luteum	Solenette	Lusitanian	3.3	15	7.2
	Callionymus reticulatus	Reticulate dragonet	Lusitanian	3.3	11	0.1
	Lumpenus lampretaeformis	Snake blenny	Boreal	3.6	49	0.4
	Platichthys flesus	Flounder	Lusitanian	3.2	50	1.1
	Pleuronectes platessa	European plaice	Boreal	3.3	100	44.9
	Pomatoschistus minutus	Sand goby	Lusitanian	3.2	10	0.8
	Scophthalmus maximus	Turbot	Lusitanian	4	100	0.4
	Syngnathus acus	Great pipefish	Lusitanian	3.4	46	0.5
	Syngnathus rostellatus	Nilsson's pipefish	Lusitanian	3.7	17	0.2
	Trachinus draco	Greater weever fish	Lusitanian	4.2	40	0.2
	Zeugopterus punctatus	Topknot	Lusitanian	4	25	0.1
Increasing	*Callionymus maculatus*	Spotted dragonet	Lusitanian	3.3	16	0.9
	Chelidonichthys cuculus	Red gurnard	Lusitanian	3.8	50	2.5
	Chelidonichthys lucerna	Tub gurnard	Lusitanian	3.7	75	0.1
	Entelurus aequoreus	Snake pipefish	Lusitanian	3.5	60	1.3
	Merluccius merluccius	European hake	Lusitanian	4.4	120	2.2
	Microchirus variegatus	Thickback sole	Lusitanian	3.3	35	0.1
	Mustelus asterias	Starry smoothhound	Lusitanian	3.7	140	0.4
	Raja brachyura	Blonde ray	Lusitanian	4	120	0.1
	Raja montagui	Spotted ray	Lusitanian	3.7	80	0.7
	Scyliorhinus canicula	Lesser spotted dogfish	Lusitanian	3.7	80	4.3
	Trisopterus minutus	Poor cod	Lusitanian	3.8	26	30.8
Decreasing	*Amblyraja radiata*	Starry ray	Boreal	4	90	8.5
	Anarhichas lupus	Wolffish (catfish)	Boreal	3.2	125	0.1
	Cyclopterus lumpus	Lumpsucker	Boreal	3.9	61	0.3
	Gadus morhua	Cod	Boreal	4.4	190	37.9
