# Supplementary material for: Community ecology in 3D: Tensor decomposition reveals spatio-temporal dynamics of large ecological communities
Source: PLoS One. 2017 Nov 14;12(11):e0188205. doi: 10.1371/journal.pone.0188205 (PMC5685633; doi:10.1371/journal.pone.0188205)
Supplement: S2 Table — Table of Pearson and RV correlation coefficient (c) with p-value (p) and adjusted p-value (ap). (PDF) [file pone.0188205.s002.pdf]

S3 Table. Full correlation coefficient table. Table of Pearson and RV correlation coefficient (c) with p-value (p) and adjusted p-value (ap).

	Tensor Decomposition												Clusters																	
	PT1			PT2			PT3			PT4			Southern			Northern			NW Inc			SE Inc			Increasing			Decreasing		
	c	pv	apv	c	pv	apv	c	pv	apv	c	pv	apv	c	pv	apv	c	pv	apv	c	pv	apv	c	pv	apv	c	pv	apv	c	pv	apv
SST_An	0.80	0.02	0.10 .	0.18	0.43	0.62	0.17	0.45	0.65	0.03	0.96	0.97	0.57	0.07	0.21	0.54	0.03	0.16	0.57	0.08	0.23	0.8	0.01	0.07 .	0.31	0.26	0.46	0.31	0.24	0.45
SST_Q1	0.47	0.10	0.28	0.24	0.29	0.47	0.4	0.14	0.34	0.03	0.91	0.95	0.25	0.29	0.47	0.21	0.40	0.60	0.73	0.03	0.16	0.93	0.0016	0.038 *	0.44	0.13	0.32	0.07	0.76	0.87
SST_var	0.51	0.08	0.23	0.26	0.27	0.46	0.28	0.23	0.44	0.02	0.87	0.92	0.24	0.28	0.47	0.27	0.26	0.46	0.65	0.05	0.17	0.96	0.0006	0.031 *	0.49	0.10	0.27	0.05	0.81	0.89
SBT_An	0.73	0.02	0.13	0.13	0.41	0.60	0.02	0.76	0.87	0.02	0.82	0.89	0.86	0.0020	0.043 *	0.23	0.29	0.47	0.46	0.11	0.28	0.28	0.2308	0.44	0.03	0.72	0.86	0.68	0.006	0.071 .
SBT_Q1	0.64	0.04	0.16	0.39	0.15	0.35	0.05	0.78	0.88	0.05	0.74	0.87	0.32	0.18	0.40	0.56	0.03	0.16	0.39	0.17	0.37	0.89	0.0012	0.031 *	0.59	0.06	0.19	0.13	0.55	0.73
SBT_var	0.94	0.0012	0.031 *	0.01	0.95	0.96	0.03	0.66	0.83	0.03	0.69	0.85	0.81	0.010	0.073 .	0.45	0.08	0.24	0.61	0.04	0.17	0.67	0.03	0.16	0.06	0.59	0.76	0.46	0.10	0.28
SSS_An	0.45	0.10	0.28	0.31	0.20	0.41	0.14	0.40	0.60	0.04	0.70	0.85	0.18	0.36	0.57	0.3	0.20	0.41	0.46	0.11	0.28	0.86	0.01	0.07 .	0.53	0.08	0.23	0.03	0.84	0.90
SSS_Q1	0.68	0.02	0.13	0.20	0.37	0.58	0.14	0.51	0.70	0.1	0.62	0.78	0.4	0.15	0.35	0.49	0.05	0.17	0.49	0.11	0.28	0.84	0.01	0.07 .	0.39	0.15	0.35	0.18	0.47	0.67
SSS_var	0.08	0.76	0.87	0.41	0.14	0.34	0.31	0.27	0.46	0.18	0.54	0.73	0.09	0.73	0.87	0.07	0.76	0.87	0.2	0.51	0.70	0.39	0.14	0.34	0.48	0.04	0.17	0.27	0.25	0.45
SBS_An	0.83	0.003	0.055 .	0.08	0.54	0.72	0.02	0.79	0.88	0.02	0.78	0.88	0.53	0.05	0.18	0.75	0.008	0.071 .	0.28	0.23	0.44	0.64	0.04	0.16	0.22	0.30	0.48	0.28	0.23	0.44
SBS_Q1	0.84	0.005	0.071 .	0.06	0.60	0.76	0.02	0.78	0.88	0.03	0.73	0.87	0.56	0.05	0.17	0.73	0.009	0.071 .	0.29	0.22	0.44	0.61	0.04	0.16	0.18	0.34	0.54	0.32	0.19	0.41
SBS_var	0.90	0.0008	0.031 *	0.13	0.52	0.71	0.05	0.90	0.94	0.04	0.94	0.96	0.67	0.02	0.14	0.55	0.05	0.17	0.54	0.07	0.21	0.79	0.010	0.073 .	0.25	0.29	0.47	0.36	0.18	0.40
Chl_An	0.88	0.0024	0.048 *	0.10	0.48	0.67	0.02	0.80	0.89	0	1.00	1.00	0.98	0.0006	0.031 *	0.31	0.22	0.44	0.54	0.06	0.19	0.38	0.14	0.34	0.01	0.85	0.91	0.72	0.015	0.097 .
Chl_Q1	0.92	0.0004	0.031 *	0.08	0.59	0.76	0.02	0.87	0.92	0	1.00	1.00	0.97	0.0048	0.071 .	0.35	0.16	0.35	0.56	0.04	0.17	0.45	0.11	0.28	0.01	0.91	0.94	0.68	0.04	0.17
Chl_var	0.83	0.006	0.071 .	0.15	0.41	0.60	0.04	0.77	0.88	0.02	0.96	0.97	0.97	0.0010	0.031 *	0.26	0.25	0.45	0.55	0.05	0.17	0.34	0.17	0.37	0.04	0.79	0.88	0.74	0.02	0.13
Fishing Otter	0.52	0.04	0.16	0.31	0.20	0.41	0.12	0.59	0.76	0.23	0.31	0.50	0.19	0.23	0.44	0.88	0.03	0.16	0.05	0.81	0.89	0.46	0.04	0.17	0.47	0.03	0.16	0.05	0.55	0.73
Fishing Beam	0.64	0.03	0.16	0.01	0.87	0.92	0.06	0.68	0.84	0.05	0.71	0.86	0.52	0.08	0.23	0.28	0.15	0.35	0.52	0.03	0.16	0.55	0.04	0.16	0.08	0.57	0.74	0.2	0.29	0.47
Beam+Otter	0.02	0.81	0.89	0.24	0.27	0.46	0.31	0.20	0.41	0.07	0.56	0.73	0.09	0.59	0.76	0.17	0.40	0.60	0.24	0.29	0.47	0.02	0.82	0.89	0.17	0.36	0.56	0.06	0.63	0.79
Climate AMO	0.40	0.03	0.16	0.40	0.04	0.16	0.4	0.04	0.16	0.74	0.009	0.071 .	0.7	0.009	0.071 .	-0.72	0.011	0.073 .	0.74	0.010	0.071 .	0.74	0.009	0.071 .	0.74	0.006	0.071 .	-0.74	0.006	0.071 .
Climate NAO	0.17	0.20	0.41	0.17	0.20	0.41	0.17	0.20	0.41	-0.06	0.40	0.60	-0.01	0.49	0.68	0.03	0.46	0.65	-0.06	0.39	0.60	-0.06	0.40	0.60	-0.06	0.39	0.60	0.06	0.41	0.60
Depth	0.88	0.008	0.071 .	0.36	0.42	0.61	-0.27	0.56	0.73	-0.3	0.51	0.70	0.66	0.10	0.28	-0.96	0.0007	0.031 *	-0.38	0.41	0.60	0.75	0.05	0.17	-0.52	0.23	0.44	-0.5	0.25	0.45
