# Supplementary material for: Community ecology in 3D: Tensor decomposition reveals spatio-temporal dynamics of large ecological communities
Source: PLoS One. 2017 Nov 14;12(11):e0188205. doi: 10.1371/journal.pone.0188205 (PMC5685633; doi:10.1371/journal.pone.0188205)

S1 Fig. Results of the Principal Tensor Analysis. Output of the *PTA-k* R-package (top) and selection of the four principal tensors (PTs) based on the scree-plot (bottom).

	PTA- 3 modes; data : 65 31 7					
	Percent Rebuilt- : 56.37922 %					
	Percent Rebuilt from Selected : 43.26678 %					
Name	PTA-k names	no	Sing Val	ssX	local Pct	Global Pct
PT1	vs111	1	57.63	14040.00	23.66	23.66
	65 vs111 31 7	3	9.10	3464.06	2.39	0.59
	65 vs111 31 7	4	5.21	3464.06	0.78	0.19
PT2	31 vs111 65 7	6	35.03	6511.03	18.85	8.74
PT3	31 vs111 65 7	7	28.10	6511.03	12.13	5.62
	7 vs111 65 31	9	17.69	4600.42	6.80	2.23
	7 vs111 65 31	10	11.46	4600.42	2.85	0.93
PT4	vs222	11	27.14	6107.06	12.06	5.25
	65 vs222 31 7	13	4.18	789.39	2.21	0.12
	65 vs222 31 7	14	3.57	789.39	1.61	0.09
	31 vs222 65 7	16	13.77	1313.69	14.44	1.35
	31 vs222 65 7	17	12.48	1313.69	11.86	1.11
	7 vs222 65 31	19	14.08	2068.85	9.58	1.41
	7 vs222 65 31	20	13.01	2068.85	8.18	1.21
	vs333	21	13.53	3408.02	5.37	1.30
	65 vs333 31 7	23	4.36	220.69	8.63	0.14
	65 vs333 31 7	24	3.07	220.69	4.26	0.07
	31 vs333 65 7	26	7.96	336.79	18.81	0.45
	31 vs333 65 7	27	6.38	336.79	12.07	0.29
	7 vs333 65 31	29	11.66	996.61	13.64	0.97
	7 vs333 65 31	30	9.61	996.61	9.28	0.66

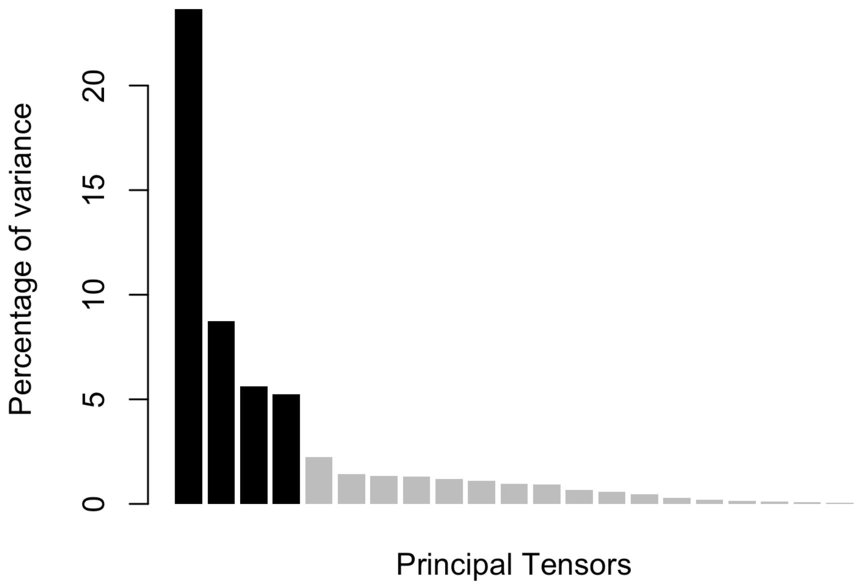

Supplement: S1 Fig — Output of the PTA-k R-package (top) and selection of the four principal tensors (PTs) based on the scree-plot (bottom). (PDF) [file pone.0188205.s003.pdf]
