# Supplementary material for: Community ecology in 3D: Tensor decomposition reveals spatio-temporal dynamics of large ecological communities
Source: PLoS One. 2017 Nov 14;12(11):e0188205. doi: 10.1371/journal.pone.0188205 (PMC5685633; doi:10.1371/journal.pone.0188205)
Supplement: S2 Fig — TD computed with (A) the method presented in the manuscript (abundance expressed in number/hour, at the scale of roundfish areas), (B) abundance expressed in biomass (catch per unit effort, expressed in kg/hour) and (C) a finer spatial resolution, at the scale of ICES rectangle (and abundance in number/hour). The three decompositions are similar, with 4 significant PT. The three first PTs show strong spatial patterns, while the PT4 shows a trend in time series. (PDF) [file pone.0188205.s004.pdf]

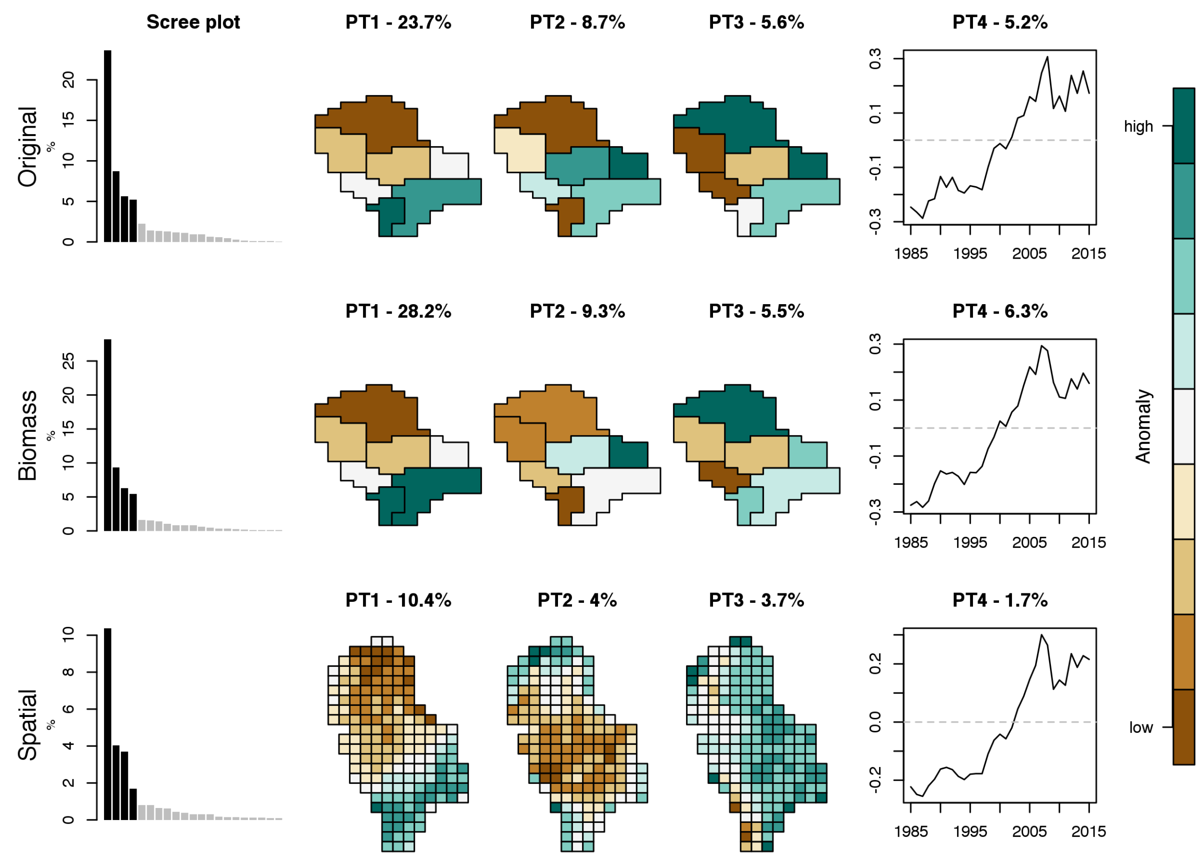

S4 Fig. Robustness analysis to data transformation and spatial scale. TD computed with (A) the method presented in the manuscript (abundance expressed in number/hour, at the scale of roundfish areas), (B) abundance expressed in biomass (catch per unit effort, expressed in kg/hour) and (C) a finer spatial resolution, at the scale of ICES rectangle (and abundance in number/hour). The three decompositions are similar, with 4 significant PT. The three first PTs show strong spatial patterns, while the PT4 shows a trend in time series.
