# Supplementary figures and images for: Community ecology in 3D: Tensor decomposition reveals spatio-temporal dynamics of large ecological communities
Source: PLoS One. 2017 Nov 14;12(11):e0188205. doi: 10.1371/journal.pone.0188205 (PMC5685633; doi:10.1371/journal.pone.0188205)

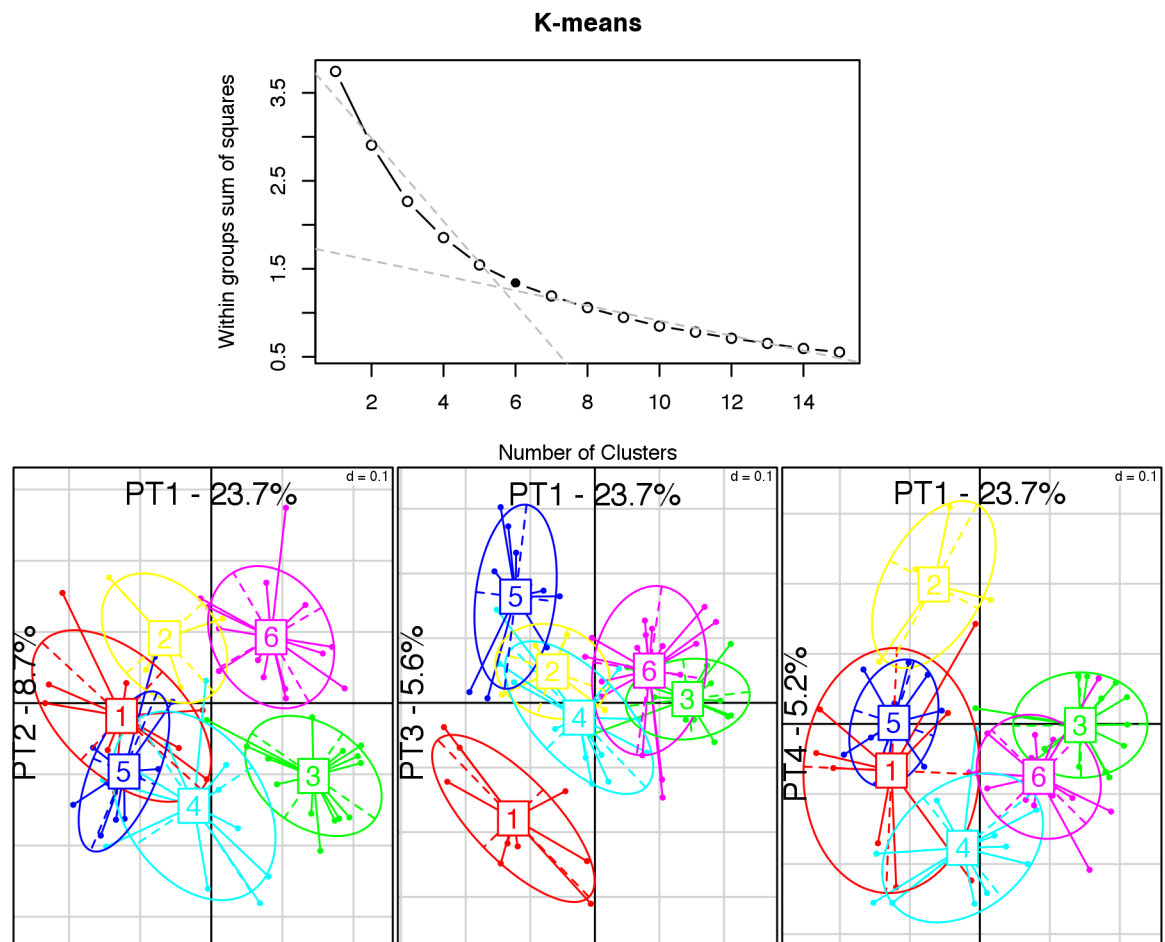

**S5 Fig. Clustering analysis of the fish species realised with K-means algorithm**

Supplement: S3 Fig — (PDF) [file pone.0188205.s005.pdf]
